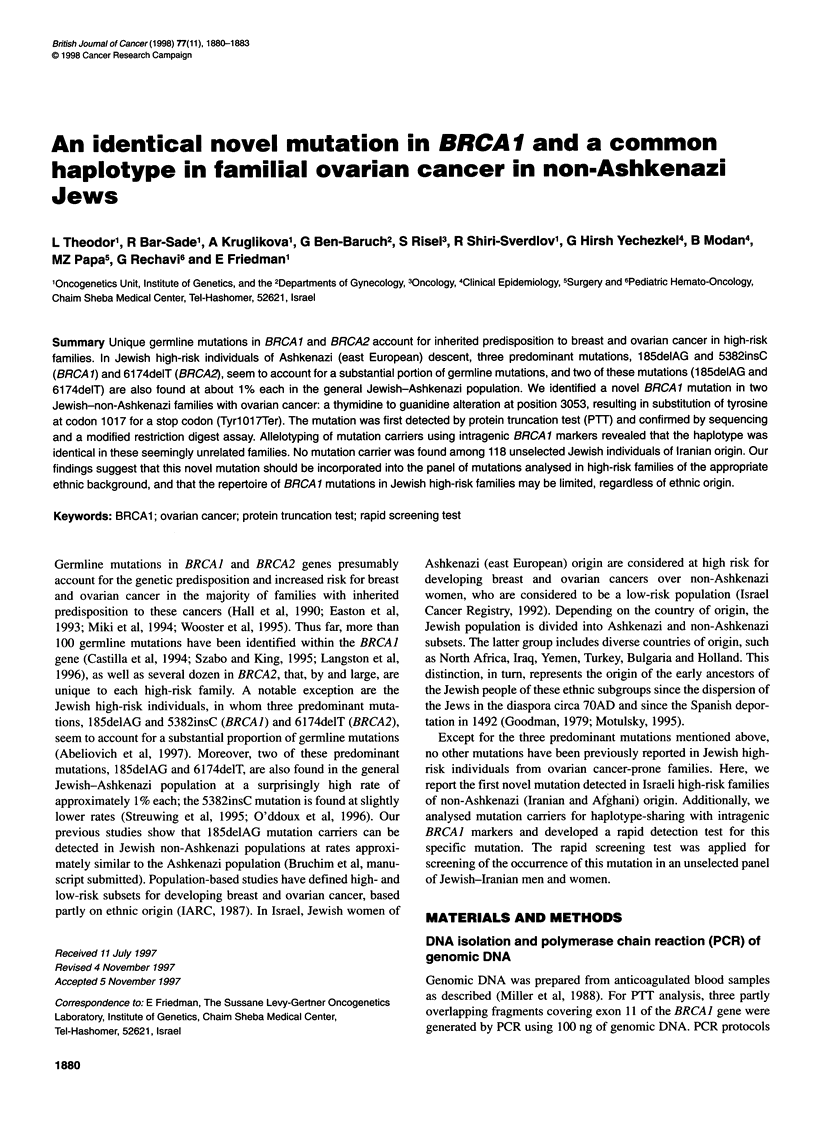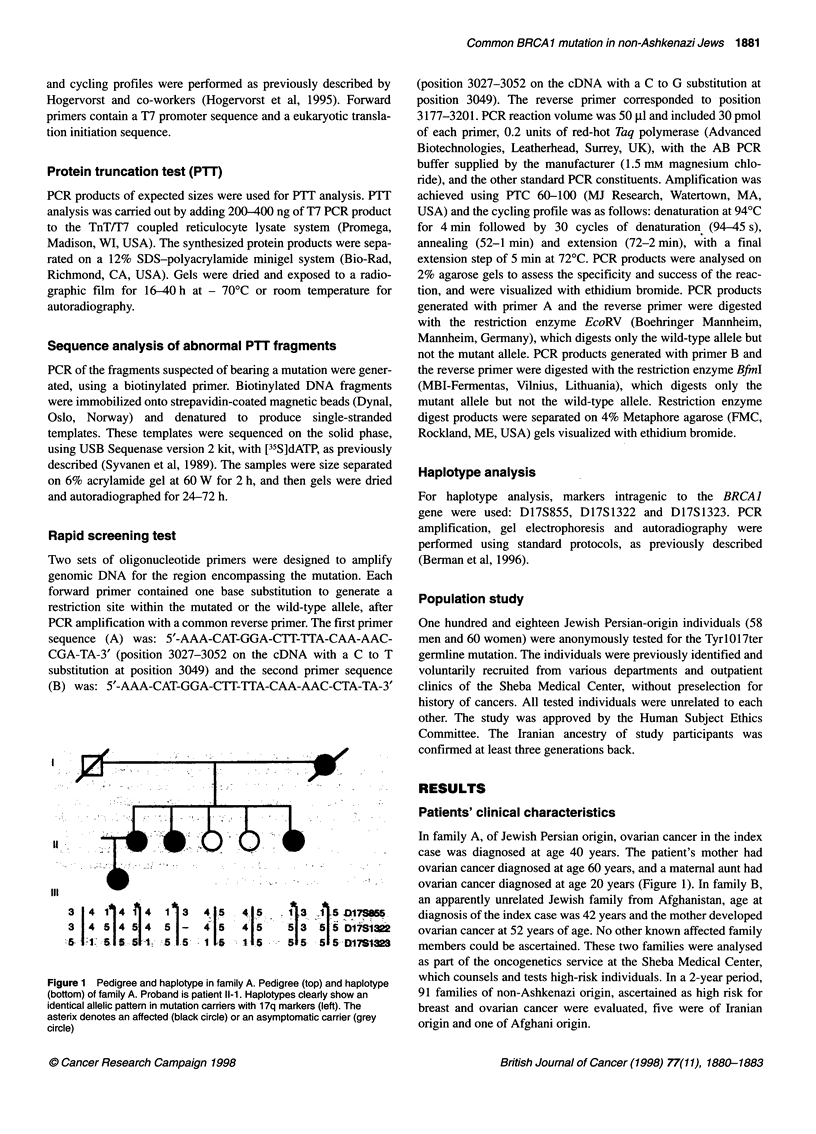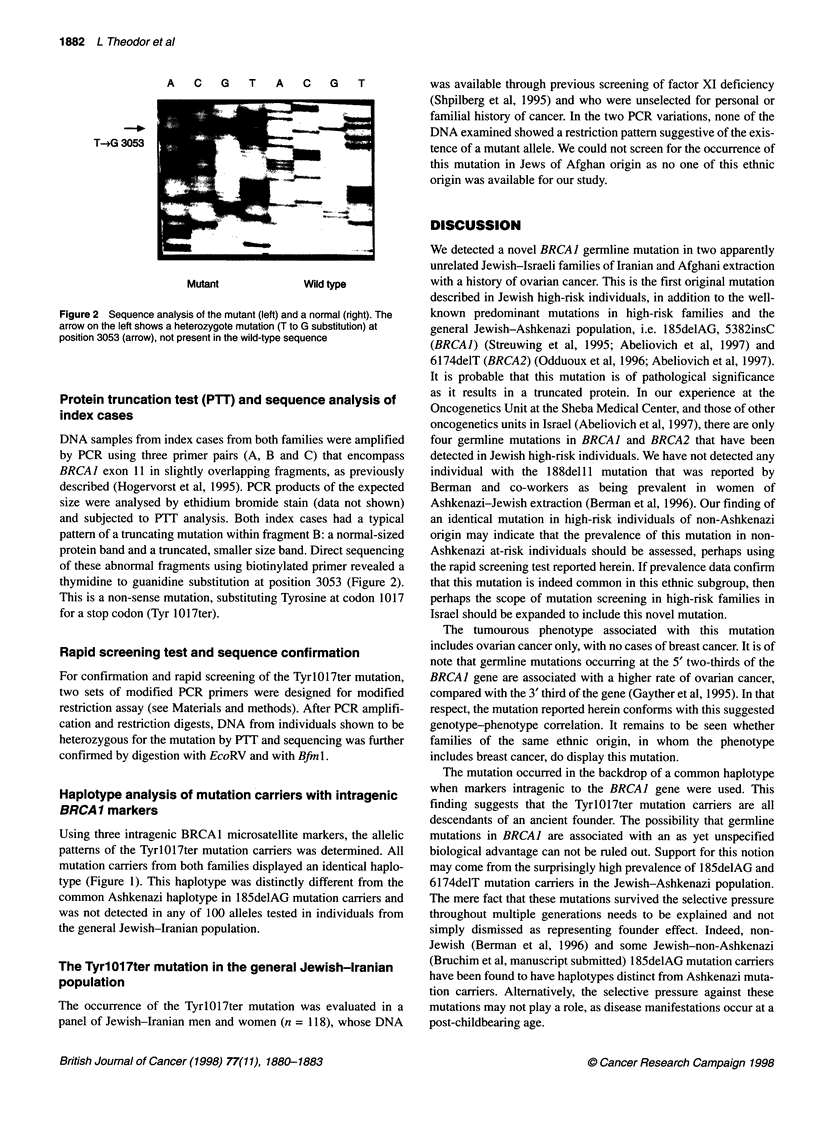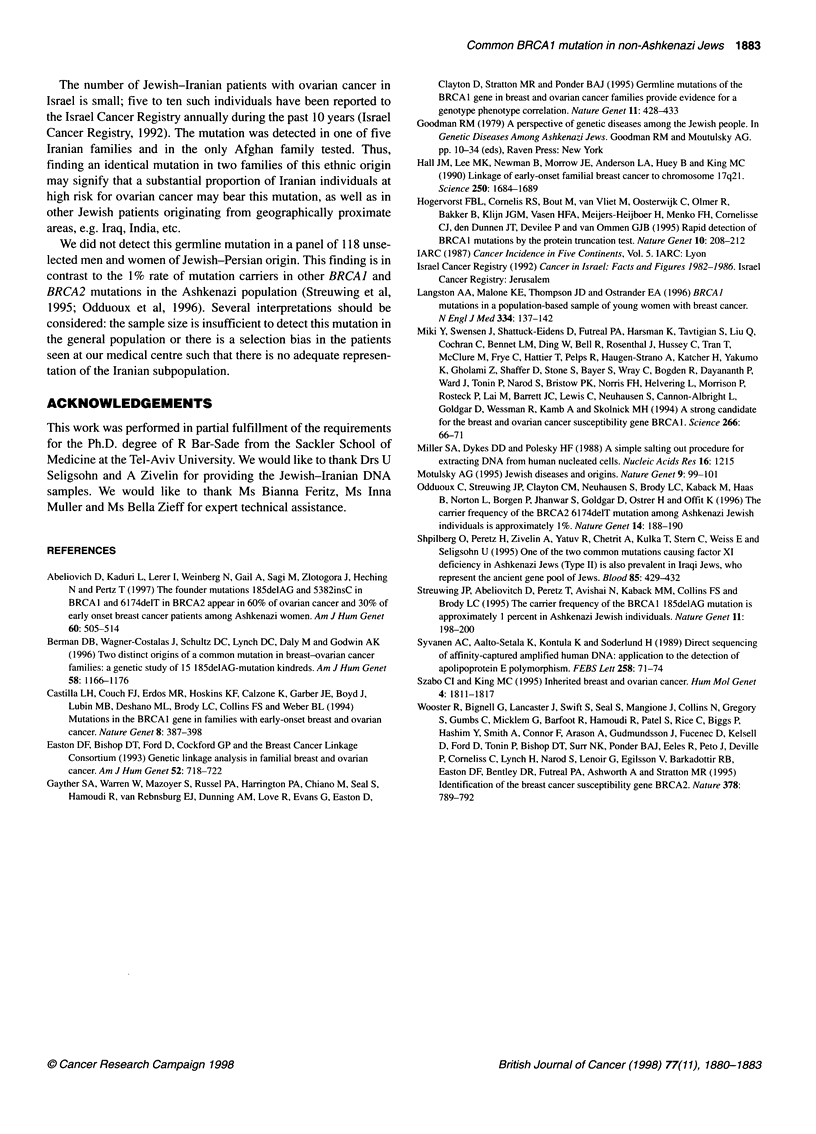# An identical novel mutation in BRCA1 and a common haplotype in familial ovarian cancer in non-Ashkenazi Jews.

**DOI:** 10.1038/bjc.1998.313

**Published:** 1998-06

**Authors:** L. Theodor, R. Bar-Sade, A. Kruglikova, G. Ben-Baruch, S. Risel, R. Shiri-Sverdlov, G. Hirsh Yechezkel, B. Modan, M. Z. Papa, G. Rechavi, E. Friedman

**Affiliations:** Oncogenetics Unit, Institute of Genetics, Chaim Sheba Medical Center, Tel-Hashomer, Israel.

## Abstract

**Images:**


*British Journal of Cancer* (1998) **77**(11), 1880–1883
© 1998 Cancer Research Campaign

### An identical novel mutation in *BRCA1* and a common haplotype in familial ovarian cancer in non-Ashkenazi Jews

**L Theodor[1], R Bar-Sade[1], A Kruglikova[1], G Ben-Baruch[2], S Risel[3], R Shiri-Sverdlov[1], G Hirsh Yechezkel[4], B Modan[4], MZ Papa[5], G Rechavi[6] and E Friedman[1]**

[1]Oncogenetics Unit, Institute of Genetics, and the [2]Departments of Gynecology, [3]Oncology, [4]Clinical Epidemiology, [5]Surgery and [6]Pediatric Hemato-Oncology, Chaim Sheba Medical Center, Tel-Hashomer, 52621, Israel

**Summary** Unique germline mutations in *BRCA1* and *BRCA2* account for inherited predisposition to breast and ovarian cancer in high-risk families. In Jewish high-risk individuals of Ashkenazi (east European) descent, three predominant mutations, 185delAG and 5382insC (*BRCA1*) and 6174delT (*BRCA2*), seem to account for a substantial portion of germline mutations, and two of these mutations (185delAG and 6174delT) are also found at about 1% each in the general Jewish–Ashkenazi population. We identified a novel *BRCA1* mutation in two Jewish–non-Ashkenazi families with ovarian cancer: a thymidine to guanidine alteration at position 3053, resulting in substitution of tyrosine at codon 1017 for a stop codon (Tyr1017Ter). The mutation was first detected by protein truncation test (PTT) and confirmed by sequencing and a modified restriction digest assay. Allelotyping of mutation carriers using intragenic *BRCA1* markers revealed that the haplotype was identical in these seemingly unrelated families. No mutation carrier was found among 118 unselected Jewish individuals of Iranian origin. Our findings suggest that this novel mutation should be incorporated into the panel of mutations analysed in high-risk families of the appropriate ethnic background, and that the repertoire of *BRCA1* mutations in Jewish high-risk families may be limited, regardless of ethnic origin.

**Keywords:** BRCA1; ovarian cancer; protein truncation test; rapid screening test

Germline mutations in *BRCA1* and *BRCA2* genes presumably account for the genetic predisposition and increased risk for breast and ovarian cancer in the majority of families with inherited predisposition to these cancers (Hall et al, 1990; Easton et al, 1993; Miki et al, 1994; Wooster et al, 1995). Thus far, more than 100 germline mutations have been identified within the *BRCA1* gene (Castilla et al, 1994; Szabo and King, 1995; Langston et al, 1996), as well as several dozen in *BRCA2*, that, by and large, are unique to each high-risk family. A notable exception are the Jewish high-risk individuals, in whom three predominant mutations, 185delAG and 5382insC (*BRCA1*) and 6174delT (*BRCA2*), seem to account for a substantial proportion of germline mutations (Abeliovich et al, 1997). Moreover, two of these predominant mutations, 185delAG and 6174delT, are also found in the general Jewish–Ashkenazi population at a surprisingly high rate of approximately 1% each; the 5382insC mutation is found at slightly lower rates (Streuwing et al, 1995; O'ddoux et al, 1996). Our previous studies show that 185delAG mutation carriers can be detected in Jewish non-Ashkenazi populations at rates approximately similar to the Ashkenazi population (Bruchim et al, manuscript submitted). Population-based studies have defined high- and low-risk subsets for developing breast and ovarian cancer, based partly on ethnic origin (IARC, 1987). In Israel, Jewish women of Ashkenazi (east European) origin are considered at high risk for developing breast and ovarian cancers over non-Ashkenazi women, who are considered to be a low-risk population (Israel Cancer Registry, 1992). Depending on the country of origin, the Jewish population is divided into Ashkenazi and non-Ashkenazi subsets. The latter group includes diverse countries of origin, such as North Africa, Iraq, Yemen, Turkey, Bulgaria and Holland. This distinction, in turn, represents the origin of the early ancestors of the Jewish people of these ethnic subgroups since the dispersion of the Jews in the diaspora circa 70AD and since the Spanish deportation in 1492 (Goodman, 1979; Motulsky, 1995).

Except for the three predominant mutations mentioned above, no other mutations have been previously reported in Jewish high-risk individuals from ovarian cancer-prone families. Here, we report the first novel mutation detected in Israeli high-risk families of non-Ashkenazi (Iranian and Afghani) origin. Additionally, we analysed mutation carriers for haplotype-sharing with intragenic *BRCA1* markers and developed a rapid detection test for this specific mutation. The rapid screening test was applied for screening of the occurrence of this mutation in an unselected panel of Jewish–Iranian men and women.

*Received 11 July 1997*
*Revised 4 November 1997*
*Accepted 5 November 1997*

*Correspondence to:* E Friedman, The Sussane Levy-Gertner Oncogenetics Laboratory, Institute of Genetics, Chaim Sheba Medical Center, Tel-Hashomer, 52621, Israel

### MATERIALS AND METHODS

### DNA isolation and polymerase chain reaction (PCR) of genomic DNA

Genomic DNA was prepared from anticoagulated blood samples as described (Miller et al, 1988). For PTT analysis, three partly overlapping fragments covering exon 11 of the *BRCA1* gene were generated by PCR using 100 ng of genomic DNA. PCR protocols and cycling profiles were performed as previously described by Hogervorst and co-workers (Hogervorst et al, 1995). Forward primers contain a T7 promoter sequence and a eukaryotic translation initiation sequence.

### Protein truncation test (PTT)

PCR products of expected sizes were used for PTT analysis. PTT analysis was carried out by adding 200–400 ng of T7 PCR product to the TnT/T7 coupled reticulocyte lysate system (Promega, Madison, WI, USA). The synthesized protein products were separated on a 12% SDS–polyacrylamide minigel system (Bio-Rad, Richmond, CA, USA). Gels were dried and exposed to a radiographic film for 16–40 h at – 70°C or room temperature for autoradiography.

### Sequence analysis of abnormal PTT fragments

PCR of the fragments suspected of bearing a mutation were generated, using a biotinylated primer. Biotinylated DNA fragments were immobilized onto strepavidin-coated magnetic beads (Dynal, Oslo, Norway) and denatured to produce single-stranded templates. These templates were sequenced on the solid phase, using USB Sequenase version 2 kit, with [$^{35}$S]dATP, as previously described (Syvanen et al, 1989). The samples were size separated on 6% acrylamide gel at 60 W for 2 h, and then gels were dried and autoradiographed for 24–72 h.

### Rapid screening test

Two sets of oligonucleotide primers were designed to amplify genomic DNA for the region encompassing the mutation. Each forward primer contained one base substitution to generate a restriction site within the mutated or the wild-type allele, after PCR amplification with a common reverse primer. The first primer sequence (A) was: 5′-AAA-CAT-GGA-CTT-TTA-CAA-AAC-CGA-TA-3′ (position 3027–3052 on the cDNA with a C to T substitution at position 3049) and the second primer sequence (B) was: 5′-AAA-CAT-GGA-CTT-TTA-CAA-AAC-CTA-TA-3′

**Figure 1** Pedigree and haplotype in family A. Pedigree (top) and haplotype (bottom) of family A. Proband is patient II-1. Haplotypes clearly show an identical allelic pattern in mutation carriers with 17q markers (left). The asterix denotes an affected (black circle) or an asymptomatic carrier (grey circle)

(position 3027–3052 on the cDNA with a C to G substitution at position 3049). The reverse primer corresponded to position 3177–3201. PCR reaction volume was 50 μl and included 30 pmol of each primer, 0.2 units of red-hot *Taq* polymerase (Advanced Biotechnologies, Leatherhead, Surrey, UK), with the AB PCR buffer supplied by the manufacturer (1.5 mM magnesium chloride), and the other standard PCR constituents. Amplification was achieved using PTC 60–100 (MJ Research, Watertown, MA, USA) and the cycling profile was as follows: denaturation at 94°C for 4 min followed by 30 cycles of denaturation (94–45 s), annealing (52–1 min) and extension (72–2 min), with a final extension step of 5 min at 72°C. PCR products were analysed on 2% agarose gels to assess the specificity and success of the reaction, and were visualized with ethidium bromide. PCR products generated with primer A and the reverse primer were digested with the restriction enzyme *Eco*RV (Boehringer Mannheim, Mannheim, Germany), which digests only the wild-type allele but not the mutant allele. PCR products generated with primer B and the reverse primer were digested with the restriction enzyme *Bfm*I (MBI-Fermentas, Vilnius, Lithuania), which digests only the mutant allele but not the wild-type allele. Restriction enzyme digest products were separated on 4% Metaphore agarose (FMC, Rockland, ME, USA) gels visualized with ethidium bromide.

### Haplotype analysis

For haplotype analysis, markers intragenic to the *BRCA1* gene were used: D17S855, D17S1322 and D17S1323. PCR amplification, gel electrophoresis and autoradiography were performed using standard protocols, as previously described (Berman et al, 1996).

### Population study

One hundred and eighteen Jewish Persian-origin individuals (58 men and 60 women) were anonymously tested for the Tyr1017ter germline mutation. The individuals were previously identified and voluntarily recruited from various departments and outpatient clinics of the Sheba Medical Center, without preselection for history of cancers. All tested individuals were unrelated to each other. The study was approved by the Human Subject Ethics Committee. The Iranian ancestry of study participants was confirmed at least three generations back.

### RESULTS

### Patients' clinical characteristics

In family A, of Jewish Persian origin, ovarian cancer in the index case was diagnosed at age 40 years. The patient's mother had ovarian cancer diagnosed at age 60 years, and a maternal aunt had ovarian cancer diagnosed at age 20 years (Figure 1). In family B, an apparently unrelated Jewish family from Afghanistan, age at diagnosis of the index case was 42 years and the mother developed ovarian cancer at 52 years of age. No other known affected family members could be ascertained. These two families were analysed as part of the oncogenetics service at the Sheba Medical Center, which counsels and tests high-risk individuals. In a 2-year period, 91 families of non-Ashkenazi origin, ascertained as high risk for breast and ovarian cancer were evaluated, five were of Iranian origin and one of Afghani origin.

© Cancer Research Campaign 1998

**Figure 2** Sequence analysis of the mutant (left) and a normal (right). The arrow on the left shows a heterozygote mutation (T to G substitution) at position 3053 (arrow), not present in the wild-type sequence

### Protein truncation test (PTT) and sequence analysis of index cases

DNA samples from index cases from both families were amplified by PCR using three primer pairs (A, B and C) that encompass *BRCA1* exon 11 in slightly overlapping fragments, as previously described (Hogervorst et al, 1995). PCR products of the expected size were analysed by ethidium bromide stain (data not shown) and subjected to PTT analysis. Both index cases had a typical pattern of a truncating mutation within fragment B: a normal-sized protein band and a truncated, smaller size band. Direct sequencing of these abnormal fragments using biotinylated primer revealed a thymidine to guanidine substitution at position 3053 (Figure 2). This is a non-sense mutation, substituting Tyrosine at codon 1017 for a stop codon (Tyr 1017ter).

### Rapid screening test and sequence confirmation

For confirmation and rapid screening of the Tyr1017ter mutation, two sets of modified PCR primers were designed for modified restriction assay (see Materials and methods). After PCR amplification and restriction digests, DNA from individuals shown to be heterozygous for the mutation by PTT and sequencing was further confirmed by digestion with *Eco*RV and with *Bfm*1.

### Haplotype analysis of mutation carriers with intragenic *BRCA1* markers

Using three intragenic BRCA1 microsatellite markers, the allelic patterns of the Tyr1017ter mutation carriers was determined. All mutation carriers from both families displayed an identical haplotype (Figure 1). This haplotype was distinctly different from the common Ashkenazi haplotype in 185delAG mutation carriers and was not detected in any of 100 alleles tested in individuals from the general Jewish–Iranian population.

### The Tyr1017ter mutation in the general Jewish–Iranian population

The occurrence of the Tyr1017ter mutation was evaluated in a panel of Jewish–Iranian men and women ($n$ = 118), whose DNA was available through previous screening of factor XI deficiency (Shpilberg et al, 1995) and who were unselected for personal or familial history of cancer. In the two PCR variations, none of the DNA examined showed a restriction pattern suggestive of the existence of a mutant allele. We could not screen for the occurrence of this mutation in Jews of Afghan origin as no one of this ethnic origin was available for our study.

### DISCUSSION

We detected a novel *BRCA1* germline mutation in two apparently unrelated Jewish–Israeli families of Iranian and Afghani extraction with a history of ovarian cancer. This is the first original mutation described in Jewish high-risk individuals, in addition to the well-known predominant mutations in high-risk families and the general Jewish–Ashkenazi population, i.e. 185delAG, 5382insC (*BRCA1*) (Streuwing et al, 1995; Abeliovich et al, 1997) and 6174delT (*BRCA2*) (Odduoux et al, 1996; Abeliovich et al, 1997). It is probable that this mutation is of pathological significance as it results in a truncated protein. In our experience at the Oncogenetics Unit at the Sheba Medical Center, and those of other oncogenetics units in Israel (Abeliovich et al, 1997), there are only four germline mutations in *BRCA1* and *BRCA2* that have been detected in Jewish high-risk individuals. We have not detected any individual with the 188del11 mutation that was reported by Berman and co-workers as being prevalent in women of Ashkenazi–Jewish extraction (Berman et al, 1996). Our finding of an identical mutation in high-risk individuals of non-Ashkenazi origin may indicate that the prevalence of this mutation in non-Ashkenazi at-risk individuals should be assessed, perhaps using the rapid screening test reported herein. If prevalence data confirm that this mutation is indeed common in this ethnic subgroup, then perhaps the scope of mutation screening in high-risk families in Israel should be expanded to include this novel mutation.

The tumourous phenotype associated with this mutation includes ovarian cancer only, with no cases of breast cancer. It is of note that germline mutations occurring at the 5′ two-thirds of the *BRCA1* gene are associated with a higher rate of ovarian cancer, compared with the 3′ third of the gene (Gayther et al, 1995). In that respect, the mutation reported herein conforms with this suggested genotype–phenotype correlation. It remains to be seen whether families of the same ethnic origin, in whom the phenotype includes breast cancer, do display this mutation.

The mutation occurred in the backdrop of a common haplotype when markers intragenic to the *BRCA1* gene were used. This finding suggests that the Tyr1017ter mutation carriers are all descendants of an ancient founder. The possibility that germline mutations in *BRCA1* are associated with an as yet unspecified biological advantage can not be ruled out. Support for this notion may come from the surprisingly high prevalence of 185delAG and 6174delT mutation carriers in the Jewish–Ashkenazi population. The mere fact that these mutations survived the selective pressure throughout multiple generations needs to be explained and not simply dismissed as representing founder effect. Indeed, non-Jewish (Berman et al, 1996) and some Jewish–non-Ashkenazi (Bruchim et al, manuscript submitted) 185delAG mutation carriers have been found to have haplotypes distinct from Ashkenazi mutation carriers. Alternatively, the selective pressure against these mutations may not play a role, as disease manifestations occur at a post-childbearing age.

© Cancer Research Campaign 1998

The number of Jewish–Iranian patients with ovarian cancer in Israel is small; five to ten such individuals have been reported to the Israel Cancer Registry annually during the past 10 years (Israel Cancer Registry, 1992). The mutation was detected in one of five Iranian families and in the only Afghan family tested. Thus, finding an identical mutation in two families of this ethnic origin may signify that a substantial proportion of Iranian individuals at high risk for ovarian cancer may bear this mutation, as well as in other Jewish patients originating from geographically proximate areas, e.g. Iraq, India, etc.

We did not detect this germline mutation in a panel of 118 unselected men and women of Jewish–Persian origin. This finding is in contrast to the 1% rate of mutation carriers in other *BRCA1* and *BRCA2* mutations in the Ashkenazi population (Streuwing et al, 1995; Odduoux et al, 1996). Several interpretations should be considered: the sample size is insufficient to detect this mutation in the general population or there is a selection bias in the patients seen at our medical centre such that there is no adequate representation of the Iranian subpopulation.

### ACKNOWLEDGEMENTS

This work was performed in partial fulfillment of the requirements for the Ph.D. degree of R Bar-Sade from the Sackler School of Medicine at the Tel-Aviv University. We would like to thank Drs U Seligsohn and A Zivelin for providing the Jewish–Iranian DNA samples. We would like to thank Ms Bianna Feritz, Ms Inna Muller and Ms Bella Zieff for expert technical assistance.

### REFERENCES

Abeliovich D, Kaduri L, Lerer I, Weinberg N, Gail A, Sagi M, Zlotogora J, Heching N and Pertz T (1997) The founder mutations 185delAG and 5382insC in BRCA1 and 6174delT in BRCA2 appear in 60% of ovarian cancer and 30% of early onset breast cancer patients among Ashkenazi women. *Am J Hum Genet* **60**: 505–514

Berman DB, Wagner-Costalas J, Schultz DC, Lynch DC, Daly M and Godwin AK (1996) Two distinct origins of a common mutation in breast–ovarian cancer families: a genetic study of 15 185delAG-mutation kindreds. *Am J Hum Genet* **58**: 1166–1176

Castilla LH, Couch FJ, Erdos MR, Hoskins KF, Calzone K, Garber JE, Boyd J, Lubin MB, Deshano ML, Brody LC, Collins FS and Weber BL (1994) Mutations in the BRCA1 gene in families with early-onset breast and ovarian cancer. *Nature Genet* **8**: 387–398

Easton DF, Bishop DT, Ford D, Cockford GP and the Breast Cancer Linkage Consortium (1993) Genetic linkage analysis in familial breast and ovarian cancer. *Am J Hum Genet* **52**: 718–722

Gayther SA, Warren W, Mazoyer S, Russel PA, Harrington PA, Chiano M, Seal S, Hamoudi R, van Rebnsburg EJ, Dunning AM, Love R, Evans G, Easton D, Clayton D, Stratton MR and Ponder BAJ (1995) Germline mutations of the BRCA1 gene in breast and ovarian cancer families provide evidence for a genotype phenotype correlation. *Nature Genet* **11**: 428–433

Goodman RM (1979) A perspective of genetic diseases among the Jewish people. In *Genetic Diseases Among Ashkenazi Jews*. Goodman RM and Moutulsky AG. pp. 10–34 (eds), Raven Press: New York

Hall JM, Lee MK, Newman B, Morrow JE, Anderson LA, Huey B and King MC (1990) Linkage of early-onset familial breast cancer to chromosome 17q21. *Science* **250**: 1684–1689

Hogervorst FBL, Cornelis RS, Bout M, van Vliet M, Oosterwijk C, Olmer R, Bakker B, Klijn JGM, Vasen HFA, Meijers-Heijboer H, Menko FH, Cornelisse CJ, den Dunnen JT, Devilee P and van Ommen GJB (1995) Rapid detection of BRCA1 mutations by the protein truncation test. *Nature Genet* **10**: 208–212

IARC (1987) *Cancer Incidence in Five Continents*, Vol. 5. IARC: Lyon

Israel Cancer Registry (1992) *Cancer in Israel: Facts and Figures 1982–1986*. Israel Cancer Registry: Jerusalem

Langston AA, Malone KE, Thompson JD and Ostrander EA (1996) *BRCA1* mutations in a population-based sample of young women with breast cancer. *N Engl J Med* **334**: 137–142

Miki Y, Swensen J, Shattuck-Eidens D, Futreal PA, Harsman K, Tavtigian S, Liu Q, Cochran C, Bennet LM, Ding W, Bell R, Rosenthal J, Hussey C, Tran T, McClure M, Frye C, Hattier T, Pelps R, Haugen-Strano A, Katcher H, Yakumo K, Gholami Z, Shaffer D, Stone S, Bayer S, Wray C, Bogden R, Dayananth P, Ward J, Tonin P, Narod S, Bristow PK, Norris FH, Helvering L, Morrison P, Rosteck P, Lai M, Barrett JC, Lewis C, Neuhausen S, Cannon-Albright L, Goldgar D, Wessman R, Kamb A and Skolnick MH (1994) A strong candidate for the breast and ovarian cancer susceptibility gene BRCA1. *Science* **266**: 66–71

Miller SA, Dykes DD and Polesky HF (1988) A simple salting out procedure for extracting DNA from human nucleated cells. *Nucleic Acids Res* **16**: 1215

Motulsky AG (1995) Jewish diseases and origins. *Nature Genet* **9**: 99–101

Odduoux C, Streuwing JP, Clayton CM, Neuhausen S, Brody LC, Kaback M, Haas B, Norton L, Borgen P, Jhanwar S, Goldgar D, Ostrer H and Offit K (1996) The carrier frequency of the BRCA2 6174delT mutation among Ashkenazi Jewish individuals is approximately 1%. *Nature Genet* **14**: 188–190

Shpilberg O, Peretz H, Zivelin A, Yatuv R, Chetrit A, Kulka T, Stern C, Weiss E and Seligsohn U (1995) One of the two common mutations causing factor XI deficiency in Ashkenazi Jews (Type II) is also prevalent in Iraqi Jews, who represent the ancient gene pool of Jews. *Blood* **85**: 429–432

Streuwing JP, Abeliovitch D, Peretz T, Avishai N, Kaback MM, Collins FS and Brody LC (1995) The carrier frequency of the BRCA1 185delAG mutation is approximately 1 percent in Ashkenazi Jewish individuals. *Nature Genet* **11**: 198–200

Syvanen AC, Aalto-Setala K, Kontula K and Soderlund H (1989) Direct sequencing of affinity-captured amplified human DNA: application to the detection of apolipoprotein E polymorphism. *FEBS Lett* **258**: 71–74

Szabo CI and King MC (1995) Inherited breast and ovarian cancer. *Hum Mol Genet* **4**: 1811–1817

Wooster R, Bignell G, Lancaster J, Swift S, Seal S, Mangione J, Collins N, Gregory S, Gumbs C, Micklem G, Barfoot R, Hamoudi R, Patel S, Rice C, Biggs P, Hashim Y, Smith A, Connor F, Arason A, Gudmundsson J, Fucenec D, Kelsell D, Ford D, Tonin P, Bishop DT, Surr NK, Ponder BAJ, Eeles R, Peto J, Deville P, Corneliss C, Lynch H, Narod S, Lenoir G, Egilsson V, Barkadottir RB, Easton DF, Bentley DR, Futreal PA, Ashworth A and Stratton MR (1995) Identification of the breast cancer susceptibility gene BRCA2. *Nature* **378**: 789–792

© Cancer Research Campaign 1998